# *Lactiplantibacillus plantarum* 06CC2 Enhanced the Expression of Intestinal Uric Acid Excretion Transporter in Mice

**DOI:** 10.3390/nu16173042

**Published:** 2024-09-09

**Authors:** Shunsuke Nei, Tatsuya Matsusaki, Hibiki Kawakubo, Kenjirou Ogawa, Kazuo Nishiyama, Chuluunbat Tsend-Ayush, Tomoki Nakano, Masahiko Takeshita, Takuo Shinyama, Masao Yamasaki

**Affiliations:** 1 Graduate School of Agriculture, University of Miyazaki, 1-1 Gakuen Kibanadai-nishi, Miyazaki 889-2192, Japan; gc19039@student.miyazaki-u.ac.jp (S.N.); t-matsusaki@dairy-milk.co.jp (T.M.); hibiki1077@icloud.com (H.K.); ogawa.kenjirou.u2@cc.miyazaki-u.ac.jp (K.O.); nishiyam@cc.miyazaki-u.ac.jp (K.N.); 2 Research and Development Division, Minami Nihon Rakuno Kyodo Co., Ltd., 5282 Takagi, Miyakonojo 885-0003, Japan; t-nakano@dairy-milk.co.jp (T.N.); m-takeshita@dairy-milk.co.jp (M.T.); t-shinyama@dairy-milk.co.jp (T.S.); 3 School of Industrial Technology, Mongolian University of Science and Technology, P.O. Box 46/520, Baga Toiruu, Sukhbaatar District, Ulaanbaatar 46, Mongolia; tsend@must.edu.mn

**Keywords:** ATP-binding cassette transporter subfamily G member 2 (ABCG2), uric acid excretion, *Lactiplantibacillus plantarum* 06CC2

## Abstract

ATP-binding cassette transporter subfamily G member 2 (ABCG2) is responsible for the excretion of foreign substances, such as uric acid (UA) and indoxyl sulfate (IS), from the body. Given the importance of increased ABCG2 expression in UA excretion, we investigated the enhancement of intestinal ABCG2 expression using *Lactiplantibacillus plantarum* 06CC2 (LP06CC2). Mice were reared on a potassium oxonate-induced high-purine model at doses of 0.02% or 0.1% LP06CC2 for three weeks. Results showed that LP06CC2 feeding resulted in increased ABCG2 expression in the small intestine. The expression level of large intestinal ABCG2 also showed a tendency to increase, suggesting upregulation of the intestinal excretion transporter ABCG2 by LP06CC2. Overall, LP06CC2 treatment increased fecal UA excretion and showed a trend towards increased fecal excretion of IS, suggesting that LP06CC2 treatment enhanced the expression of intestinal ABCG2, thereby promoting the excretion of UA and other substances from the intestinal tract.

## 1. Introduction

Hyperuricemia is a lifestyle-related metabolic disease characterized by elevated blood uric acid (UA) levels. The incidence of hyperuricemia and gout is increasing worldwide. UA is produced in the body mainly as a product of purine bases derived from nucleic acid metabolism, and 10–20% is derived from cellular purines. Although UA in the body is excreted from the kidneys and organs other than the kidneys (mainly the intestine), deterioration of the excretion system causes accumulation and crystallization of UA in the body, which leads to gout-related complications. Urate-lowering drugs such as benzbromarone and probenecid are designed to promote UA excretion via the renal pathway [1]. In this pathway, UA is synthesized from purines by xanthine oxidase (XOD). Allopurinol and febuxostat are drugs that inhibit UA production by blocking XOD [2,3]. However, these drugs have various side effects that limit their clinical use [2,4]. Therefore, it is necessary to develop effective treatment and prevention methods with fewer side effects.

Recent studies have focused on reduced extrarenal excretion in hyperuricemia, primarily the excretion of UA via the intestinal tract [5]. UA excretion is mediated by the ATP-binding cassette transporter subfamily G member 2 (ABCG2). ABCG2 belongs to the subfamily G of the ABC emission transporter superfamily and is known to be involved in the ATP-dependent transport of various substrate compounds from the intracellular to the extracellular space [6]. ABCG2 has been identified as a urate excretion factor that mediates renal and extrarenal urate excretion [7]. It is also shown to be a transporter of the uremic toxin indoxyl sulphate (IS) [8]. ABCG2 dysfunction is associated with reduced UA excretion, and patients with hyperuricemia show reduced ABCG2 expression [5]. Therefore, ABCG2 may be a putative target for the treatment or prevention of hyperuricemia. Previous reports have shown the lowering effects of lactic acid bacteria on blood UA levels, and our study explored the UA level-lowering effects of our own lactic acid bacteria. Another study reported that Lactobacillus administration increased ABCG2 expression in the gut [9]. We evaluated the effects of a lactic acid bacterial strain *Lactiplantibacillus plantarum* 06CC2 (LP06CC2) isolated from traditional Mongolian dairy products on hyperuricemia. In previous studies, LP06CC2 has shown probiotic properties [10] and anti-inflammatory effects in inflammatory bowel disease [11]. Additionally, we evaluated the expression of intestinal ABCG2 by LP06CC2 of the lactic acid bacteria. 

## 2. Materials and Methods

### 2.1. Materials

Potassium Oxonate was purchased from TCI (Tokyo, Japan). Uric acid (UA) and allopurinol were purchased from FUJIFILM Wako Pure Chemical Corporation (Osaka, Japan). Lithium carbonate was purchased from NACALAI TESQUE, INC. (Kyoto, Japan). Pterine and isoxanthopterin were purchased from SIGMA-ALDRICH (St. Louis, MO, USA). K_2_HPO_4_ was purchased from FUJIFILM Wako Pure Chemical Corporation, EDTA-2Na was purchased from DOJINDO LABORATORIES (Kumamoto, Japan). HRP secondary antibodies was purchased from Cell Signaling TECHNOLOGY (Danvers, MO, USA).

### 2.2. Preparation of LP06CC2

In a previous study, LP06CC2 (a probiotic derived from Mongolian dairy products) was reported to be resistant to bile and gastric acids and adhere to Caco-2 cells [10]. LP06CC2 was precultured at 37 °C for 18 h in de Man, Rogosa, and Sharpe (MRS) broth (Merck Millipore, Darmstadt, Germany). Next, 5.0 mL of the precultured suspension was introduced into 500 mL of MRS broth and incubated at 37 °C for 18 h. After fermentation, the optical density was measured at a wavelength of 660 nm and found to be approximately 5.0–5.5. The bacteria were centrifuged at 1500× *g* for 5 min, washed twice with phosphate-buffered saline (PBS) and lyophilized.

### 2.3. Animal Experiments

Five-week-old male BALB/c mice, weighing 18–23 g, were obtained from Japan SLC Corporation (Shizuoka, Japan) and maintained at 22 °C in a room with a 12 h light–dark cycle. After 7 days of acclimatizing, the mice were divided into four groups (n = 8): control (Con) group, high-purine diet (PO) group, high-purine diet + LP06CC2 (PO + LP) group, and high-purine diet + low-volume LP06CC2 (PO + L-LP) group. Each group was fed freely for three weeks. They were fed an AIN-93G-based normal diet (Con) or high-purine diet with potassium oxonate (PO) mixed in the diet at 25 g/kg. Potassium oxonate mixed in the feed to inhibit uric acid production. LP06CC2 was mixed in the feed at a concentration of 0.1% (6.4 × 10^7^ CFU/g) for PO + LP and 0.02% (1.2 × 10^7^ CFU/g) for PO + L-LP (Table 1). The diets were stored at −80 °C. Three animals in each group were moved to metabolic cages from six weeks of age, and five animals in each group were kept in flat cages, fed, and watered once every two days, at which time the amount of food, urine, and feces were measured. Body weights were measured once every 7 days. The feces were pooled individually and preserved at −80 °C. After anesthesia by intraperitoneal administration, blood was collected by cardiac blood sampling. Blood was centrifuged at 4 °C, 1000 rpm, 20 min, after which the supernatant was collected and cryopreserved as plasma. Liver, kidney, small intestine, large intestine and cecum were collected and homogenized (100 mg/mL) in RIPA buffer at 4 °C, 4000 rpm for 3 min, then the supernatant was collected and preserved at −30 °C.

### 2.4. Western Blot Analysis 

Organs were homogenized in RIPA buffer (FUJIFILM Wako) containing protease inhibitors (NACALAI TESQUE). Protein concentrations were determined using the BCA protein Assay Kit (Thermo Fisher Scientific, Waltham, MA, USA). Proteins were separated on 12.5% gel (SuperSepTMAce) and transferred to PVDF transfer membrane (GE Healthcare, Chicago, IL, USA). Membranes were blocked with 5% skim milk (2.0 g Morinaga skim milk dissolved in 40 mL of 0.1% T-TBS) and incubated with primary antibody overnight at 4 °C. Primary antibodies ABCG2 (Anti-BCRP, ABCG2 antibody), PDZ domain-containing protein 1 (PDZK1, Anti-PDZK1), organic anion transporter 1 (OAT1, OAT1 Polyclonal antibody) and urate transporter 1 (URAT1, URAT1 Polyclonal antibody) were purchased from Proteintech Group, Inc. (Rosemont, IL, USA). β-Actin (Monoclonal Anti-β-Actin, antibody) was purchased from SIGMA-ALDRICH. Next, the membranes were incubated with HRP secondary antibodies. HRP secondary antibodies was purchased from Cell Signaling TECHNOLOGY. The bands were immersed in ClarityMAX Western ECL Substrate (Bio-Rad Laboratories, Inc., Hercules, CA, USA) and visualized using a Lumino Image Analyzer LAS-4000 (FUJIFILM, Tokyo, Japan). The analysis software ImageQuant TL ver. 8.1 (FUJIFILM) was used to calculate the density of the bands and the results were corrected for β-Actin.

### 2.5. Measurement of UA

#### 2.5.1. UA Extraction from Feces

A suitable amount of freeze-dried feces and a piece of dry ice were placed in the cup of a crusher (Osaka Chemical Co., Ltd., Osaka, Japan) and pulverized until a powder was obtained, allowing the dry ice to sublimate completely. Next, 10 mg of crushed feces was placed in an Eppendorf tube, 500 µL of 0.4% lithium carbonate solution and 500 µL of pure water were added and incubated for 24 h at room temperature. After that, the supernatant was collected by cooling centrifugation at 12,000× *g*, 4 °C, 10 min.

#### 2.5.2. Sample Preparation

Urine: Urine samples were centrifuged at 10,000× *g* for 10 min at 4 °C, and the supernatant was diluted 10-fold with pure water.

Feces: UA-extraction solutions were used for the measurements without dilution. 

Plasma: An aliquot of plasma (30 µL) was diluted 10-fold with 270 µL of pure water. It was then cooled and centrifuged at 300× *g* for 3 min at 4 °C. The supernatant was collected and 60% perchloric acid was added at 10% of the volume recovered after cooling centrifugation. The mixture was centrifuged at 10,000× *g* for 5 min, 4 °C, and the supernatant was collected and used as the sample. All samples were filtered when transferring to the vials. 

#### 2.5.3. Measurement

An Inertsil ODS-2 column (GL Science Inc., Tokyo, Japan: 5020-01128) was used to perform HPLC (Agilent technologies, Inc. 1220 Infinity II, Santa Clara, CA, USA) separation. UA was analyzed UV-VIS detector at 284 mm. The mobile phase of this analysis was 74 mM phosphate buffer and methanol (98:2) at the flow rate 1 mL/min. Column temperature was 40 °C.

### 2.6. Measurement of XOD Activity 

50 µL of the collected protein sample and 50 µL of 100 µM pterine solution diluted in buffer (50 mM K_2_HPO_4_ and 0.1 mM EDTA-2Na stirred together) were applied. The fluorescence intensity was measured using a multifunctional microplate reader (Perkin Elmer, Waltham, MA, USA) at 355/405 nm for 0–20 min, every 2 min.

### 2.7. Measurement of Protein-Derived Gut Microbiota Metabolites in the Feces

Indoxyl sulfate (IS) in the feces was measured by the method described by Antoine et al. [12]. The lyophilized fecal samples (80 µg) were mixed with 200 µL of 10 µM 4-methylumbelliferyl sulfate (4-MS) as the internal standard and 500 µL of methanol-water (1:1). The mixture was sonicated, cooled at 4 °C for 30 min and centrifuged at 15,000× *g* for 10 min. The supernatant was evaporated and dissolved in 200 µL of methanol-water (1:1).

The samples were purified using an ultra-free MC-HV centrifugal filter unit (Merck Millipore, Burlington, MA, USA) before separation on a liquid chromatography/mass spectrometry (LC/MS) apparatus. The LC/MS analysis was performed on an ACQUITY UPLC H-class system (Waters, Milford, MA, USA), which was equipped with an ACQUITY UPLC HSS T3 column (1.8 μm, 100 mm × 2.1 mm; Waters). The mobile phase A was water, which contained 0.1% formic acid. The mobile phase B consisted of acetonitrile containing 0.1% formic acid. Gradient elution with a linearly increasing concentration gradient of acetonitrile was employed at a flow rate of 0.3 mL/min. The pump was programmed as follows: In the first 7 min, 5% mobile phase B was maintained. Mobile phase B was increased from 5% to 50% for 11 min, 50% to 100% for 2 min, and maintained for another 5 min. A 2 μL portion of the sample was conditioned at 4 °C and injected into the system within 8 h. Next, the column temperature was maintained at 50 °C. Electrospray ionization mass spectrometry (ESI-MS) was performed in the negative ion mode, and the capillary voltage was adjusted to 1.0 kV.

### 2.8. Measurement of Short-Chain Fatty Acids in the Cecal Contents and Feces

Short-chain fatty acids (SCFAs) were measured by LC/MS as previously described [13,14]. Briefly, lyophilized fecal samples (80 mg) were suspended in 15 mL of water and centrifuged at 8000× *g* for 10 min. The supernatant was collected and used for SCFA analysis. The supernatant (100 μL) was mixed with 100 μL of 500 μM 2-ethylbutyrate as the internal standard, 200 μL of 20 mM 2-nitrophenylhydrazine–HCl (2-NPH), and 400 μL of 1-(3-dimethylaminopropyl)-3-ethylcarbodiimide-HCl. Thereafter, it was heated for 20 min at 60 °C. Next, 100 μL of 15% potassium hydroxide in methanol-water (4:1) was added, and the mixture was heated for 15 min at 60 °C. Furthermore, 2-NPH-labeled SCFAs were extracted twice with diethyl ether. Subsequently, the extracts were evaporated and the residue was dissolved in methanol for LC/MS analysis using an ACQUITY UPLC HSS T3 column (Waters^TM^, Milford, MA, USA). Elution was performed with a linearly increasing concentration gradient of acetonitrile, with mobile phases consisting of water containing 0.05% formic acid (mobile phase A) and acetonitrile containing 0.05% formic acid (mobile phase B), at a flow rate of 0.15 mL/min. The pump was programmed as follows: In the first 5 min, 20% mobile phase B was maintained. Mobile phase B was increased from 20% to 60% over 15 min and maintained for 2 min. A 1.0 μL portion of the sample was conditioned at 10 °C and injected into the system within 8 h. Next, the column temperature was maintained at 50 °C. ESI-MS was performed in the positive ion mode, and the capillary voltage was adjusted to 3.0 kV.

### 2.9. Statistical Analysis 

Results analysis was performed using statistical software, Statcel 4 4th edition (OMS Publishing, Saitama, Japan). Significant differences between groups were examined using the Tukey–Kramer method for testing each parameter. A risk rate (*p*-value) of less than 0.05 or less than 0.01 was considered significantly different. Values are expressed as mean ± standard error. Simple regression analysis was tested using the same software and a risk rate (*p*-value) of less than 0.05 or less than 0.01 was considered a significant difference.

## 3. Results

### 3.1. Growth Parameters and Tissue Weights

Table 2 shows various growth parameters after 3 weeks of feeding. Results showed that there were no significant differences in body weight, food intake and urine output among dietary groups. Various organ weight data are shown in Table 3. A significant increase in small intestine weight was observed in the PO and PO + L-LP groups compared to the Con group.

### 3.2. UA and Liver XOD Activity

**Figure 1:**
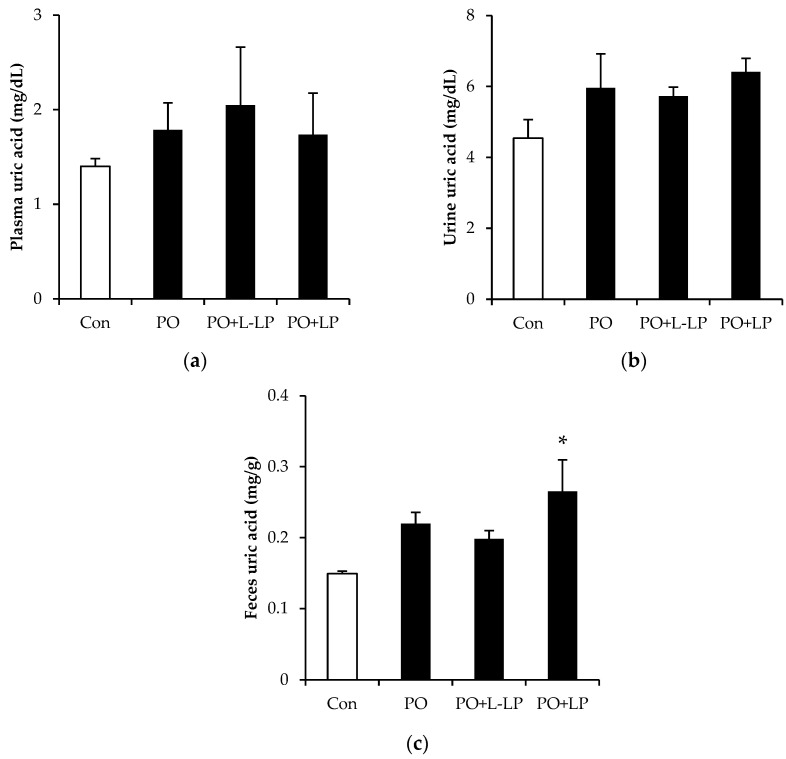
The effects of *Lactiplantibacillus plantarum* 06CC2 on plasma (**a**), urine (**b**), and feces (**c**) uric acid levels Data are the means ± SE, n = 8, Significant difference * *p* < 0.05 vs. Control for Tukey–Kramer. Con: control diet, PO: potassium oxonate diet, PO + LP: potassium oxonate, 0.1% LP06CC2 diet, PO + L-LP: potassium oxonate, and 0.02% (low) LP06CC2 diet, SE: standard error.

Figure 1 shows UA levels in the plasma, urine, and feces. There were no significant differences in the plasma and urinary UA levels among the dietary groups, whereas the fecal UA levels were significantly higher in the PO + LP group than in the control group. 

**Figure 2:**
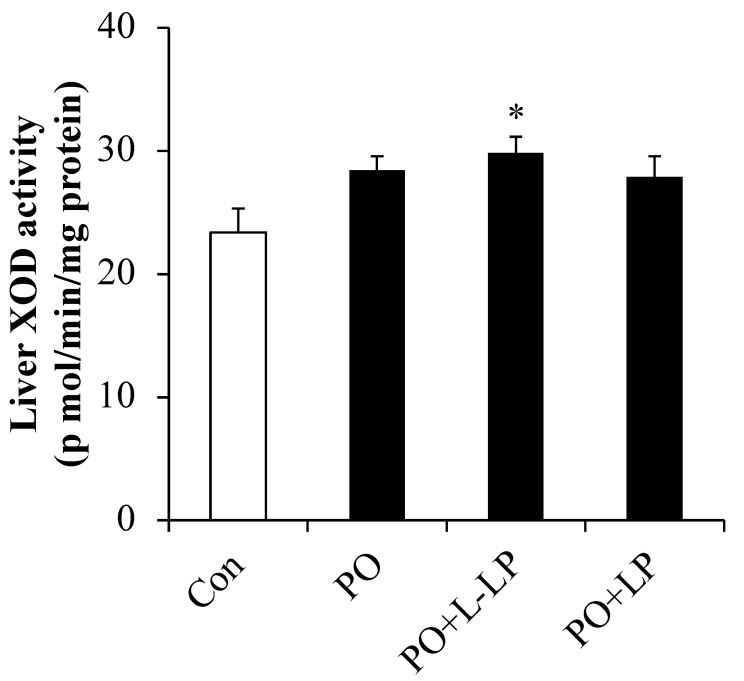
Liver XOD activity. Effect of *Lactiplantibacillus plantarum* 06CC2 on xanthine oxidase activity in the liver Data are the means ± SE, n = 8, Significant difference * *p* < 0.05 vs. Control for Tukey–Kramer. Con: control diet, PO: potassium oxonate diet, PO + LP: potassium oxonate, 0.1% LP06CC2 diet, PO + L-LP: potassium oxonate, and 0.02% (low) LP06CC2 diet, SE: standard error.

XOD (xanthine oxidase) activity in the liver is presented in Figure 2. XOD activity was significantly higher in the PO + L-LP group than that in the control group.

### 3.3. Intestinal UA Excretion Transporter Expression Level

**Figure 3:**
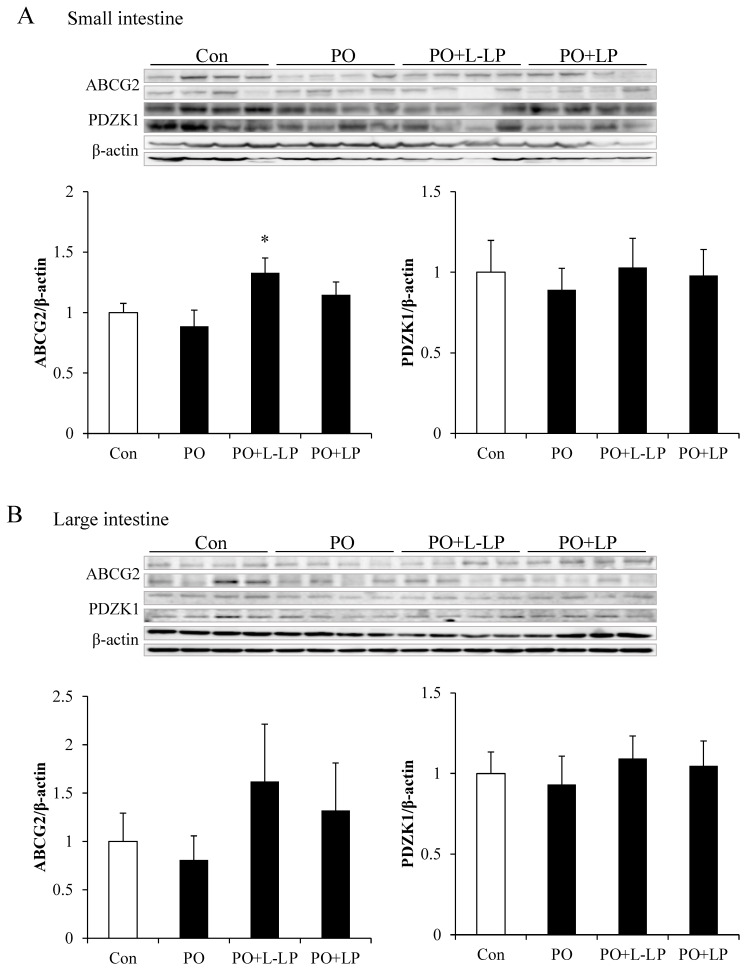
Intestinal uric acid excretion transporter expression level. Effect of *Lactiplantibacillus plantarum* 06CC2 on uric acid excretion transporter expression levels in the intestine ABCG2 and PDZK1 transporter expression in the small intestine (**A**). ABCG2 and PDZK1 transporter expression in the large intestine (**B**). Data are the means ± SE, n = 8, * *p* < 0.05 vs. PO for the Tukey–Kramer test. Con: control diet, PO: potassium oxonate diet, PO + LP: potassium oxonate, 0.1% LP06CC2 diet, PO + L-LP: potassium oxonate, and 0.02% (low) LP06CC2 diet, SE: standard error.

Figure 3 shows the expression levels of intestinal UA excretion transporters. As UA excretion transporters, ABCG2 and PDZK1 are scaffold proteins that mediate subcellular localization of the ABCG2 transporter. The expression of the ABCG2 transporter was significantly higher in the PO + L-LP group than that in the PO group. No significant differences in PDZK1 expression were observed between dietary groups. 

No significant differences were observed in the expression levels in the large intestine between the groups. However, small- and large-intestinal ABCG2 expression levels were found to be increased at lower rather than higher LP06CC2 volumes.

### 3.4. UA Excretion Transporter Expression Level in Kidney

**Figure 4:**
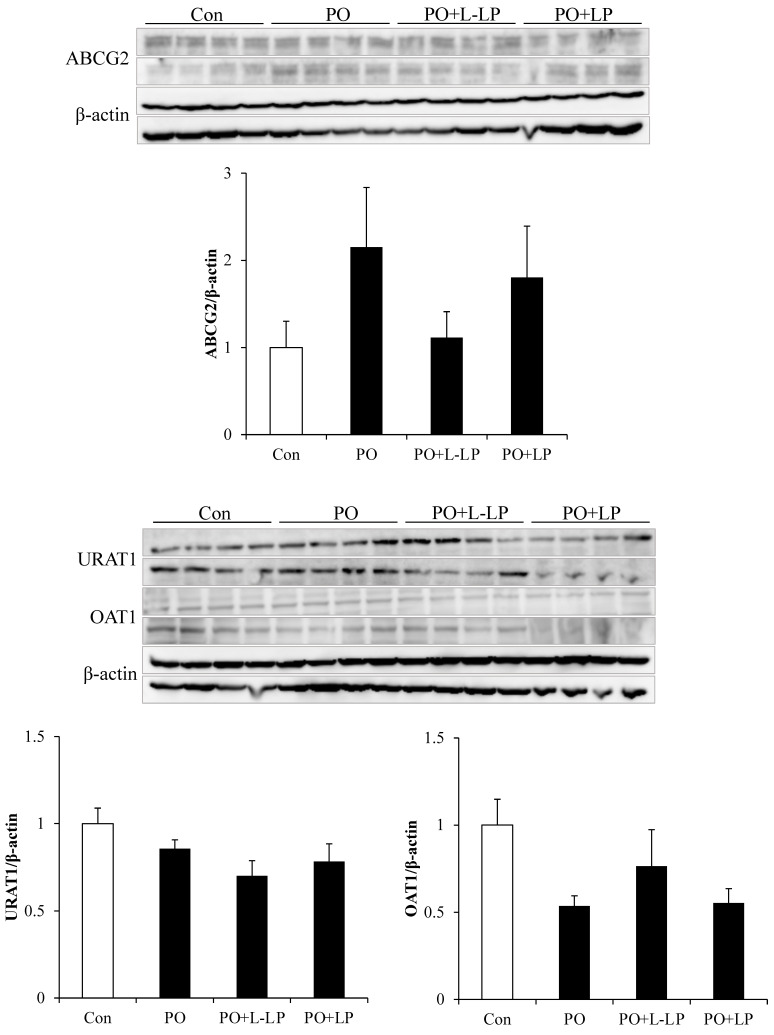
Uric acid excretion transporter expression level in kidney. Effect of *Lactiplantibacillus plantarum* 06CC2 on uric acid excretion transporter expression levels in the kidney Expression levels of ABCG2, URAT1 and OAT1 transporters in kidneys. Data are the means ± SE, n = 8. Con: control diet, PO: potassium oxonate diet, PO + LP: potassium oxonate, 0.1% LP06CC2 diet, PO + L-LP: potassium oxonate, and 0.02% (low) LP06CC2 diet, SE: standard error.

Expression levels of UA excretion transporters in the kidneys are shown in Figure 4. 

In the kidney, UA is transported from the blood into the renal proximal tubular epithelial cells via the OAT1 and excreted into the urine via ABCG2. To maintain UA levels in the body, URAT1 plays a role in the reabsorption of UA on the luminal sides of the tubules. There were no significant differences in transporter expression levels in the kidneys among the different dietary groups. However, the expression level of OAT1 was downregulated in the PO group and increased with LP06CC2 treatment. ABCG2 expression was upregulated in the PO group.

### 3.5. Fecal Excretion of Protein-Derived Gut Microbiota Metabolites

**Figure 5:**
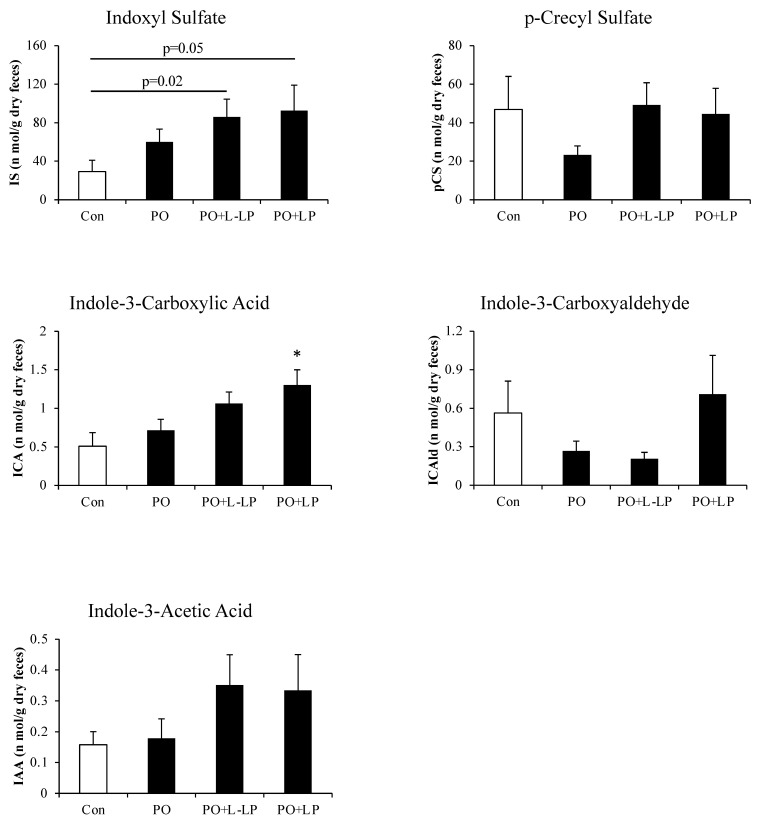
Fecal excretion of protein-derived gut microbiota metabolites. Effect of *Lactiplantibacillus plantarum* 06CC2 on the fecal excretion of protein-derived gut microbiota metabolites. Data are the means ± SE, n = 8, * *p* < 0.05 vs. PO for the Tukey–Kramer test. Con: control diet, PO: potassium oxonate diet, PO + LP: potassium oxonate, 0.1% LP06CC2 diet, PO + L-LP: potassium oxonate, and 0.02% (low) LP06CC2 diet, SE: standard error.

Expression levels of Protein-Derived Gut Microbiota Metabolites are shown in Figure 5.

ABCG2 transports the uremic toxin IS out of the body as a substrate. As the expression of intestinal ABCG2 was increased by LP06CC2 treatment, the actual excretion of IS in the feces was measured.

Fecal excretion of Indole-3-Carboxylic Acid was significantly higher in the PO + LP group than in the Con group. There were no significant differences in the fecal excretion of IS, but excretion increased in the PO group compared with that in the Con group and tended to increase in a concentration-dependent manner with LP06CC2 administration. Con vs. PO + LP (*p* = 0.05), Con vs. PO + L-LP (*p* = 0.02).

### 3.6. Fecal and Cecal SCFAs Content

**Figure 6:**
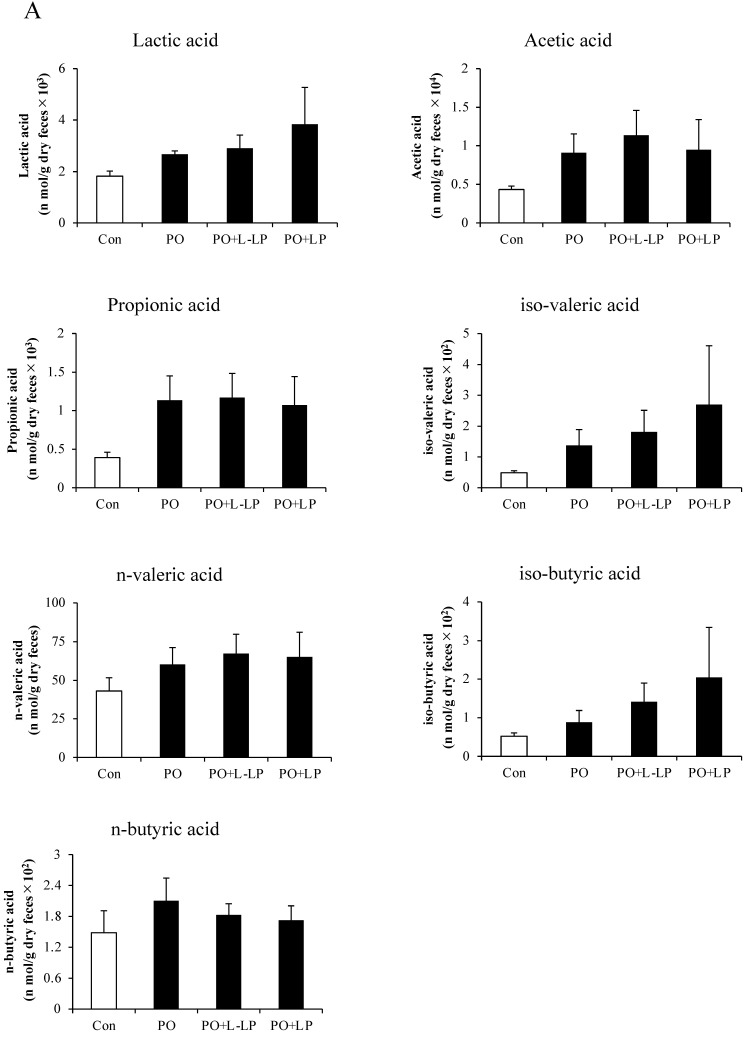

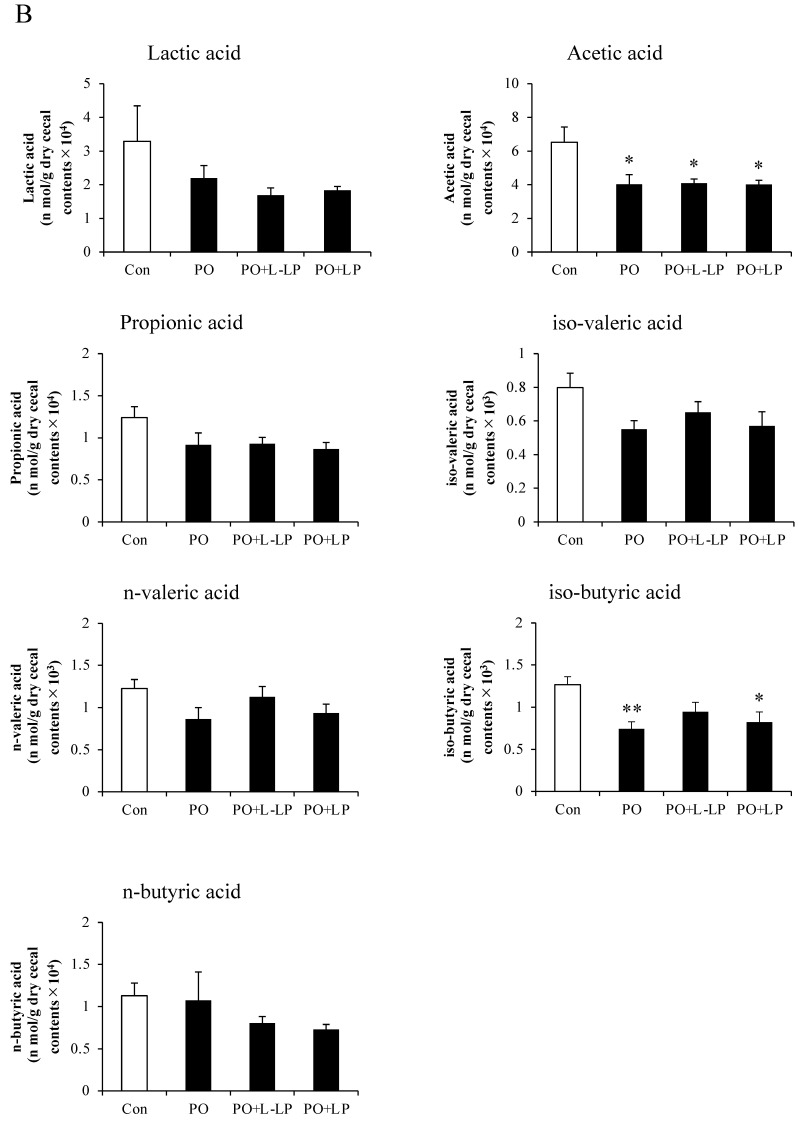
Short-chain fatty acids. Effect of *Lactiplantibacillus plantarum* 06CC2 on short-chain fatty acids (SCFAs) in fecal excretion (**A**) and cecal content (**B**). Data are the means ± SE, n = 8, ** *p* < 0.01, * *p* < 0.05 vs. PO for the Tukey–Kramer test. Con: control diet, PO: potassium oxonate diet, PO + LP: potassium oxonate, 0.1% LP06CC2 diet, PO + L-LP: potassium oxonate, and 0.02% (low) LP06CC2 diet, SE: standard error.

Figure 6 shows the results of Lactic acid and SCFAs content in the feces (A) and cecum (B).

No significant differences in Lactic acid and SCFAs production were observed in the feces after LP06CC2 treatment. However, cecal levels of acetic acid were significantly lower in the PO, PO + LP, and PO + L-LP groups than in the Con group. A significant decrease in isobutyric acid was observed in the PO and PO + L-LP groups.

### 3.7. Correlation between Fecal UA Levels and Fecal Indoxyl Sulphate

**Figure 7:**
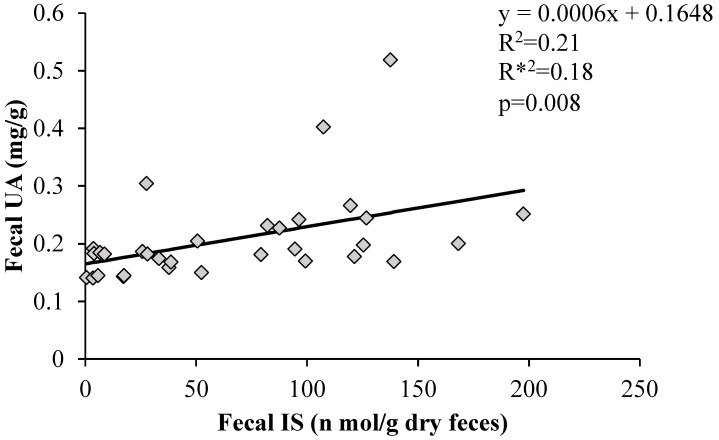
Correlation between fecal uric acid levels and fecal indoxyl sulphate. Graph of the correlation coefficients between fecal uric acid and fecal indoxyl sulfate levels using Statcel 4. R^2^: coefficient of determination; R*^2^: degrees of freedom adjusted coefficient of determination; *p*: *p*-value, UA: uric acid, IS: indoxyl sulfate.

As IS and UA are excreted into the intestinal tract via ABCG2, we examined whether IS and UA levels in the feces were correlated. Figure 7 shows the correlation between fecal UA and IS excretion. A significant positive correlation was found between the two factors.

## 4. Discussion

Hyperuricemia is a lifestyle-related disease in which UA levels in the body increase and accumulate in the joints and other parts of the body, causing UA crystallization and resulting in gout. Two-thirds of the UA produced in the body by metabolism through diet and exercise is excreted by the kidney, and one-third by organs other than the kidneys, such as the intestine [15]. UA in the body is balanced by its synthesis and excretion [15]. The kidney has been recognized as the primary site of serum UA regulation in humans [5]. Drugs such as benzbromarone and probenecid are designed to promote UA excretion via the renal pathway [1]. However, the current research indicates that the intestine is an important organ in UA excretion [16]. In previous studies, the UA-lowering effects of Lactobacillus included a decrease in xanthine oxidase (XOD) activity, which is involved in UA synthesis [17], and promotion of UA excretion from the kidneys [18,19,20]. Conversely, LP06CC2 increased liver XOD activity after L-LP administration, but LP06CC2 did not lead to increased plasma UA levels. Urinary excretion of UA also showed no difference after LP06CC2 administration, indicating that the results did not involve kidney-mediated UA excretion.

There are several reports in which potassium oxonate was used to induce hyperuricemia via intraperitoneal administration [21,22]. We applied this model via oral administration by mixing it with diet to continuously maintain high blood UA concentrations. As show in Figure 1, compared with intraperitoneal administration, the increase in blood UA appeared to be moderate.

In the present study, LP06CC2 administration with PO was found to increase the expression of the ABCG2 transporter in the small and large intestines. In addition, increased UA excretion in the feces was observed. However, intestinal ABCG2 expression was highest in the low-dose LP06CC2 group, whereas UA excretion in feces was highest in the high-dose LP06CC2 group. These results suggest that there is an optimal capacity for LP06CC2 upregulation of ABCG2 expression. It has also been reported that intestinal UA is rapidly metabolized by bacterial flora in the intestinal lumen, and that intestinal UA excretion is difficult to assess [5]. Therefore, it was difficult to determine whether LP06CC2 promotes UA excretion via ABCG2 mediated pathway.

It has also been reported that ABCG2 excretes not only UA but also IS [8]. As shown in Figure 5, LP06CC2 moderately promoted fecal excretion of IS. Furthermore, the fecal excretion of other dietary protein-derived components tended to increase with LP06CC2 treatment. IS is mainly excreted from the kidneys via urine. Although no difference in UA urinary excretion was observed in the present high-purine diet mouse model, kidney ABCG2 expression showed an increasing trend in the PO group and a decreasing trend in LP06CC2 group. Conversely, intestinal ABCG2 expression increased, suggesting that LP06CC2 promotes IS intestinal excretion, which may be inversely correlated with renal excretion. Previous studies have shown that intestinal ABCG2 expression is inversely correlated with blood and urinary UA levels [8]. A positive correlation was also observed between fecal UA and IS excretion. The promotion of the intestinal excretion of UA by LP06CC2 suggests that fecal excretion of IS is promoted via intestinal ABCG2.

To determine the mechanisms underlying the upregulation of intestinal ABCG2 expression, we evaluated fecal and cecal short-chain fatty acid (SCFA) levels. A previous study reported that the addition of short-chain fatty acids to Caco-2 cells, an intestinal epithelial cell line, increased ABCG2 expression [23]. We previously showed that SCFA levels were significantly increased by LP06CC2 treatment [13]. However, no increase in SCFAs was observed in the feces or cecum in the present study. This discrepancy may be because the previous studies were performed using higher doses of LP06CC2 than the current dose of 0.1% LP06CC2 treatment [13,14]. LP06CC2 doses of 0.1% or 0.02%, which were the doses used in this study, did not result in an increase in SCFA production, and the effect of increased intestinal ABCG2 expression was unlikely to be influenced by SCFAs.

## 5. Conclusions

In summary, treatment with Lactobacillus LP06CC2 increased the expression of intestinal ABCG2 transporters, particularly in the small intestine. Increased expression of intestinal ABCG2 may be effective for eliminating UA from the body and alleviating the symptoms of hyperuricemia, suggesting that LP06CC2 may provide a new strategy for the treatment or prevention of hyperuricemia. The molecular mechanism underlying the increase in ABCG2 expression induced by LP06CC2 remains unclear. In addition, single dose of PO intraperitoneally or oral gavage is generally applied for the establishment of acute induction of hyperuricemia. Here, PO was mixed with diet to collect feces under chronic uricase inhibition. Repeated injection of PO slightly increased fecal excretion of UA [24] and our data showed similar tendency. But plasma UA levels failed to increase in the mice used in this study, and it is important to evaluate their effects on hyperuricemia in the future.

## Figures and Tables

**Table 1:** Dietary composition (g/kg).

Ingredients (g)	Con	PO	PO + L-LP	PO + LP
Casein	200	200	200	200
Corn starch	397.5	362.5	362.3	361.5
α-Corn starch	132	132	132	132
Sucrose	100	100	100	100
Soybean oil	70	70	70	70
Cellulose	50	50	50	50
Choline bitartrate	2.5	2.5	2.5	2.5
L-cystine	3	3	3	3
Mineral mix	35	35	35	35
Vitamin mix	10	10	10	10
*tert*-Butylhydroquinone	0.014	0.014	0.014	0.014
RNA from yeast	0	10	10	10
Potassium oxonate	0	25	25	25
LP06CC2	0	0	0.2	1
Total	1000.014	1000.014	1000.014	1000.014

Con: control diet, PO: potassium oxonate diet, PO + LP: potassium oxonate and 0.1% LP06CC2 diet, PO + L-LP: potassium oxonate and 0.02% (low) LP06CC2 diet.

**Table 2:** Growth parameters.

	Con	PO	PO + L-LP	PO + LP
Final body weight (g)	24.1 ± 0.39	23.5 ± 0.25	23.3 ± 0.56	23.2 ± 0.45
Food intake (g/day)	3.11 ± 0.09	3.20 ± 0.11	3.40 ± 0.17	3.01 ± 0.07
Urine volume (mL/day)	1.01 ± 0.06	1.17 ± 0.11	0.81 ± 0.11	0.91 ± 0.05

Data shown are given as mean ± SE, n = 8 (final body weight and food intake). Urine volume was measured from mice in metabolic cages (n = 3). Con: control diet, PO: potassium oxonate diet, PO + LP: potassium oxonate and 0.1% LP06CC2 diet, PO + L-LP: potassium oxonate and 0.02% (low) LP06CC2 diet, SE: standard error.

**Table 3:** Organ weights.

(mg/g Body Weight)	Con	PO	PO + L-LP	PO + LP
Liver	47.2 ± 0.8	47.5 ± 0.9	46.9 ± 1.2	46.3 ± 0.6
Epididymal fat	14.0 ± 0.8	11.9 ± 0.8	11.9 ± 1.7	13.0 ± 0.9
Small intestine	19.8 ± 1.2	29.6 ± 1.3	29.1 ± 1.5	24.7 ± 1.9
Large intestine	6.3 ± 0.2	6.6 ± 0.2	6.9 ± 0.2	6.3 ± 0.3
Kidney	18.0 ± 0.3	18.2 ± 0.5	18.0 ± 0.6	17.2 ± 0.2
Perinephric fat	2.7 ± 0.2	2.4 ± 0.2	2.2 ± 0.2	2.4 ± 0.3

Data are the means ± SE, n = 8. Con: control diet, PO: potassium oxonate diet, PO + LP: potassium oxonate and 0.1% LP06CC2 diet, PO + L-LP: potassium oxonate and 0.02% (low) LP06CC2 diet, SE: standard error.

## Data Availability

The original contributions presented in the study are included in the article, further inquiries can be directed to the corresponding author.
